# The Impact of Coronary Artery Bypass Grafting on Respiratory Function: A Systematic Review

**DOI:** 10.3390/jcm15072793

**Published:** 2026-04-07

**Authors:** Gonçalo Flores, Pedro Duarte-Mendes, Hélder Fonseca, Diogo Monteiro, Fernanda M. Silva, Nuno Couto, Ana Maria Silva, João Paulo Vilas-Boas

**Affiliations:** 1 Faculty of Sport, University of Porto, 4099-002 Porto, Portugal; goncalofloresft@outlook.com (G.F.); hfonseca@fade.up.pt (H.F.); 2 Research Center in Physical Activity Health and Leisure (CIAFEL), Faculty of Sport, University of Porto, 4099-002 Porto, Portugal; 3 Department of Sports and Well-Being, Polytechnic Institute of Castelo Branco, 6000-084 Castelo Branco, Portugal; 4 Sport Physical Activity and Health Research & Innovation Center (SPRINT), 2040-413 Santarém, Portugal; 5 School of Education and Social Sciences, Polytechnic University of Leiria, 2411-901 Leiria, Portugal; diogo.monteiro@ipleiria.pt; 6 Research Centre in Sport, Health and Human Development (CIDESD), 5001-801 Vila Real, Portugal; ncouto@esdrm.ipsantarem.pt; 7 School of Education and Communication, University of Algarve, 8005-139 Faro, Portugal; geral.fernandasilva@gmail.com; 8 Research Unit for Sport and Physical Activity (CIDAF), University of Coimbra, 3040-248 Coimbra, Portugal; 9 Sport Sciences School of Rio Maior, Santarém Polytechnic University, 2040-413 Santarém, Portugal; 10 Department of Life Sciences, University of Coimbra, 3000-456 Coimbra, Portugal; amgsilva@antrop.uc.pt; 11 Center for Research in Anthropology and Health (CIAS), 3000-456 Coimbra, Portugal; 12 Research Centre for Sports Research, Education, Innovation and Intervention in Sport, CIFI2D, Faculty of Sport, University of Porto, 4099-002 Porto, Portugal; jpvb@fade.up.pt; 13 Porto Biomechanics Laboratory (LABIOMEP-UP), University of Porto, 4099-002 Porto, Portugal

**Keywords:** pulmonary function, respiratory muscle strength, health outcomes, coronary artery bypass surgery, cardiac surgery

## Abstract

**Background**: Cardiovascular diseases are the main cause of mortality and morbidity in Portugal, with coronary artery bypass grafting (CABG) being one of the most performed surgeries in cardiothoracic centers. After cardiac surgery, patients often experience a decrease in physical capacity, which results in an increased risk of mortality or hospitalization expenditures. The objective of this systematic review was to characterize changes in respiratory function in patients undergoing CABG. **Methods**: This systematic review followed the Preferred Reporting Items for Systematic Reviews and Meta-Analysis. Web of Science, Pubmed, SCOPUS, and Sport Discus were searched using a predefined research strategy to identify relevant original studies published until August 2025. To be included, studies must have assessed adult patients submitted to CABG who evaluated the respiratory function before and after cardiac surgery. Studies that reported other types of cardiac surgery were excluded. The Risk of Bias in Non-randomized Studies-of-Exposure and the Cochrane risk-of-bias tool for randomized trials were used to analyze the risk of bias of the selected studies. **Results**: After screening 1184 potential articles, six studies met the inclusion criteria. The studies included participants who underwent CABG (n = 324), with a mean age ranging from 54.05 ± 13.6 to 67 ± 10 years. **Conclusions**: All included studies reported significant postoperative reductions in respiratory function following CABG, including forced vital capacity, forced expiratory volume in one second, maximal inspiratory pressure, and maximal expiratory pressure. Although these findings consistently indicate a decline in pulmonary function, the limited number of available studies limits the strength of the conclusions. This systematic review suggests that monitoring respiratory impairments after CABG may be clinically relevant to improve health-related quality of life.

## 1. Introduction

Cardiovascular diseases have been increasing worldwide and are the major cause of mortality and morbidity in Portugal [1]. In this context, coronary artery bypass grafting (CABG) is among the most frequently performed surgeries in cardiothoracic canters [1,2], as surgical treatment remains the best therapeutic option for improved survival [3].

During the initial weeks after cardiac surgery, many patients experience a decrease in physical capacity and autonomic nervous system disfunction [4], which can increase hospitalizations and mortality risk [5,6]. It is also frequent for these patients to experience a significant reduction in quality of life, due to impairment of psychosocial well-being promoting stress and depression [7], which can result in increased hospital costs [3].

Respiratory dysfunction after coronary artery bypass grafting is very common and significantly contributes to impairments in physical capacity, being one of the major causes of morbidity and mortality after CABG [8]. Respiratory function can be evaluated using a manometer to measure respiratory muscle strength by measuring maximal inspiratory pressure (MIP) and maximal expiratory pressure (MEP) at the mouth [9,10]. In addition, air volume and airflow rates can be assessed through spirometry, with forced vital capacity (FVC) and forced expiratory volume in one second (FEV_1_) being the most relevant parameters in patients undergoing cardiac surgery [10].

To the best of our knowledge, no systematic review or meta-analysis is available that examines the evidence on the impact of CABG on respiratory function. Respiratory impairment after CABG can contribute to postoperative complications, which contribute to a delay in recovery and a reduction in health-related quality of life [5,6]. Given the prevalence and clinical impact of these alterations, a synthesis of the available evidence is crucial to clarify the magnitude of the respiratory changes following cardiac surgery. This knowledge is essential for guiding clinical decisions and developing recovery strategies, considering their clinical importance for patient prognosis following cardiac surgery. Therefore, this systematic review aims to investigate the changes in respiratory function in patients undergoing coronary artery bypass grafting. We hypothesized that coronary artery bypass grafting is associated with a significant postoperative reduction in respiratory function.

## 2. Material and Methods

This systematic review followed the Preferred Reporting Items for Systematic Reviews and Meta-analysis (PRISMA) guidelines (Table S1) [11]. The protocol was registered with the PROSPERO International Prospective Register of Systematic Reviews under the registration number: CRD420251086180.

### 2.1. Eligibility Criteria

Studies that met the following criteria defined by the PECOS framework [12] were included in this systematic review (Table 1): (1) studies that included participants submitted to CABG; (2) studies that evaluated respiratory function before and after cardiac surgery; (3) studies that reported data for individuals over 18 years of age; (4) and studies published until August 2025. Studies reporting other types of cardiac surgery were excluded from this systematic review.

### 2.2. Search Strategy

The search for this systematic review was conducted in the following databases: Web of Science, Pubmed, SCOPUS, and Sport Discus, accessed between 15 June 2025, and 7 August 2025. The search strategy included all types of study designs using descriptors to filter the data: (“cardiac rehabilitation” OR “heart surgery” OR “coronary heart disease” OR “cardiac surgery” OR “coronary artery bypass grafting” OR “GABG”) AND (“respiratory function” OR “respiratory muscle strength” OR “muscle strength” OR “inspiratory muscle training”) AND (“adult”). This was supplemented by manually reviewing the reference lists of the selected articles to identify all potentially relevant studies.

### 2.3. Selection Process and Data Extraction

Studies initially identified in the database searches were imported to EndNote 21 (Clarivate, Philadelphia) and duplicates were removed automatically. In the first phase, two reviewers (GF and PM) independently searched for relevant studies in the initial selection, based on title and abstract (n = 1149 screened). In the second phase, the full texts of the selected studies were reviewed according to the defined eligibility criteria (n = 11) [13]. In case of disagreement, the decision to include or not to include the study was resolved through involvement of a third reviewer (DM). This process of studies selection was made independently by two authors (GF and PM). Data were extracted independently by two reviewers (GF and PM) using a piloted extraction form, collecting information on study characteristics (name of the authors, sample size, sex, age, methodological design, timing of assessment, respiratory function outcomes and main conclusions).

### 2.4. Study Risk of Bias Assessment

The Risk of Bias in Non-randomized Studies-of-Exposure (ROBINS-E) was used independently by two reviewers to evaluate the quality and risk of bias of the observational studies identified as eligible. This scale addresses seven domains: confounding, measurement of exposure, selection of participants, post-exposure interventions, missing data, measurement of outcomes, and selective reporting of results [14]. In each study, each domain can be rated as “low”, “high”, or “some concerns” [14].

To assess the risk of bias for each randomized controlled trial, the Cochrane risk-of-bias tool for randomized trials (ROB 2) was applied independently by two investigators. The ROB 2 evaluates five domains: the randomization process, deviations from intended interventions, missing outcome data, and measurement of the outcome and selection of the reported result [15]. In each randomized controlled trial, the assessment can be categorized as “Low” or “High” risk of bias, or it can indicate “Some concerns” [15].

## 3. Results

**Figure 1:**
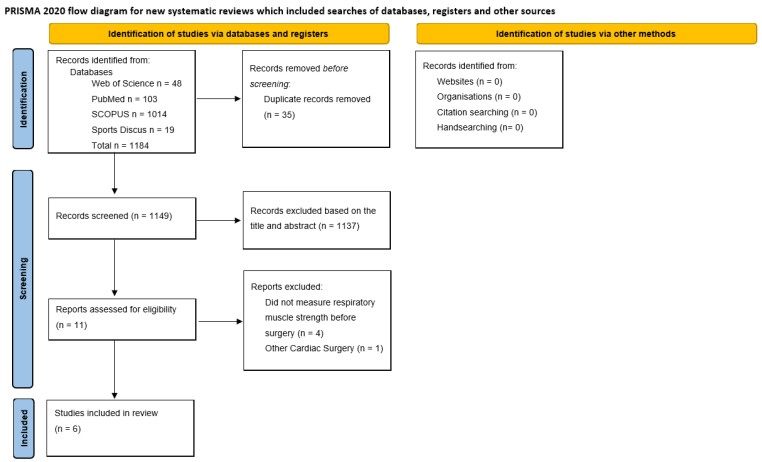
Search strategy flowchart.

The sequence followed for selecting the studies included in this systematic review is shown in the search strategy flowchart (Figure 1). The initial literature search identified a total of 1184 potentially eligible studies. After excluding studies based on duplicates (35), titles and abstracts (1149), 12 full-text articles were examined according to inclusion and exclusion criteria. Afterwards, six studies were excluded for the reasons presented in Figure 1, leaving six for analysis.

Details of the six studies included in this systematic review are presented in Table 2. The six studies included patients submitted to elective CABG for chronic coronary syndrome (n = 324), with a mean age ranging between 54.05 ± 13.6 and 67 ± 10 years. The review included two randomized controlled trials and four cohort studies, with patients of both sexes (75% males and 25% females). The study from Johnson et al. [16] does not report the patients’ sex. All study assessments were made in local hospitals; specifically, the studies by Cordeiro et al. [3] and Riedi et al. [17] were conducted in Brazil, Naseer et al. [9] in Saudi Arabia, Sacvi et al. [18] in Turkey, and Urell et al. [10] in Sweden. Sacvi et al. [18] performed the CABG with saphenous vein grafts and the left internal mammary artery and Urell et al. [10] made a median sternotomy. Other included studies did not specify the cardiac surgery procedure.

Cordeiro et al. [3], Johnson et al. [16], Sacvi et al. [18] and Riedi et al. [17] evaluated patients before surgery and again before discharge (one week after surgery). Naseer et al. [9], Johnson et al. [16] and Urell et al. [10] assessed patients before surgery and two months after surgery. All studies excluded emergency cardiac surgery. Cordeiro et al. [3] used an analogic manometer to assess respiratory muscle strength and considered the best result of three evaluations. Johnson et al. [16] used an electronic spirometer (Spirometer #61000; Welsh Allan, Pneumocheck, Skaneatles Falls, NY, USA) and a manometer (Boehringer Inspiratory Force Meter #4100; Norristown, PA, USA) with the patients sitting upright with a pillow against their chest to minimize pain. Riedi et al. [17] used an analogic manometer (Wika MV300 model) and the test was performed with the patients sitting with the lower limbs extended and feet resting on the ground. The test was performed three times, and the best value was considered for analysis. Naseer et al. [9] assessed patients using a spirometry device and a manometer (undisclosed equipment) in a seated position and performed the test three times, reporting the best value. Sacvi et al. [18] used a spirometry device (Spirolab, Medical International Research, Rome, Italy) and a portable electronic mouth pressure device (Micro MPM, Micro Medical Ltd., Kent, UK) to test pulmonary function in a seated position, considering the best value of the three performed evaluations. Urell et al. [10] also used a spirometry tool (Jaeger MasterScreenPFT/Bodybox—Intramedic, Bålsta, Sweden) and a manometer in a seated position to assess respiratory function.

Johnson et al. [16] reported postoperative complications, including cough (n = 15), phlegm (n = 11), wheeze (n = 6), dyspnea (n = 6), and episodes of bronchitis (n = 1). Riedi et al. [17] reported postoperative complications, including cough and dyspnea (n = 5), bronchospasm, hypoxemia, or atelectasis (n = 12), pneumonia, pneumothorax, or pleural effusion (n = 6), and ventilation failure or intubation (n = 3).

Urell et al. [10] demonstrated that two months after cardiac surgery there was a decrease in MIP (73 ± 22 cmH_2_O) and a significant decrease in MEP (115 ± 38 cmH_2_O) compared to the preoperative values (78 ± 24 and 122 ± 33 cmH_2_O, respectively). This study similarly verified that FEV_1_ was significantly reduced in patients submitted to cardiac surgery (from 3.0 ± 0.8 to 2.8 ± 0.7 L) [10]. These results are in line with Naseer et al. [9], who demonstrated a significant reduction in FEV_1_ after cardiac surgery compared with preoperative values (from 2.87 ± 0.45 to 2.5 ± 0.68 L) [9]. This study also demonstrated reductions in MIP (from 81.75 ± 22.04 to 74.56 ± 18.86 cmH_2_O) and MEP (from 98.55 ± 22.24 to 88.86 ± 18.14 cmH_2_O) after cardiac surgery. Johnson et al. [16] showed that FVC (from 3.51 ± 0 82 to 2 91± 0 72 L), FEV_1_ (2.74 ± 0.74 L) and MEP (from 93 ± 28 to 89 ± 26 cmH_2_O) values all reduced in patients submitted to CABG. Savci et al. [18] also demonstrated a significant decrease in pulmonary function between preoperative values and discharge, more precisely in FVC (from 85.00 ± 13.71 to 66.43 ± 14.42% in the control group and from 88.00 ± 16.36 to 64.00 ± 14.94% in the intervention group), FEV_1_ (from 77.24 ± 14.59 to 64.29 ± 14.90% in the control group and from 85.95 ± 16.75 to 63.73 ± 15.06% in the intervention group), and MEP (from 101.71 ± 22.22 to 73.43 ± 25.52 cmH_2_O in the control group and from 106.55 ± 33.27 to 69.82 ± 14.60 cmH_2_O in the intervention group). A reduction in MEP (from 89.18 ± 30.18 to 66.8 ± 22.11 cmH_2_O) and MIP (from 106.2 ± 49.42 to 91.5 ± 52.2 cmH_2_O) values after cardiac surgery was also observed by Riedi et al. [17] compared to preoperative values, and a significant reduction in MIP was observed in Cordeiro et al. [3] (from 97.5 ± 18.2 to 69.5 ± 14.9 cmH_2_O in the control group and from 94.2 ± 16.2 to 83.1 ± 19.1 cmH_2_O in the intervention group) and Savci et al. [18] (from 84.62 ± 17.26 to 57.24 ± 19.48 in the control group).

As mentioned, the four included observational studies were assessed using the ROBINS-E (Table 3), while the two randomized controlled trials were evaluated using the ROB-2 (Table 4). The randomized controlled studies included were rated as having a “low risk” of bias, and the observational studies as having a “Moderate” risk of bias.

## 4. Discussion

The objective of this systematic review was to assess the impact of CABG on respiratory function. The results demonstrated impairments in maximal inspiratory and expiratory pressures, forced vital capacity, and forced expiratory volume in one second following coronary artery bypass grafting, with moderate-to-large effect sizes for most variables, suggesting that respiratory function impairment may be clinically relevant during the early postoperative period.

After cardiac surgery, respiratory dysfunction is one of the most frequent causes of morbidity and mortality, potentially leading to longer hospital stays and higher costs associated with the surgery [3,19]. Cargnin et al. [20] reported an association between reduced respiratory muscle function and the development of postoperative pulmonary complications, although this study analyzed patients submitted to heart valve replacement surgery. Similarly, Hermes et al. [5] reported that respiratory muscle dysfunction in patients undergoing CABG may contribute to delayed pulmonary recovery by reducing functional capacity.

Cordeiro et al. [3] proposed that the systemic inflammatory response triggered by cardiac surgery is associated with alterations in pulmonary function, including reduced lung compliance, pulmonary edema, and/or decreased functional residual capacity. These changes were associated with a postoperative reduction in MIP in both groups, which was more significant in the control group (Cohen’s *d* = −1.68), which followed the normal post-surgical care routine of the unit (undisclosed protocol). In the training group, MIP also decreased, but with a moderate effect size (Cohen’s *d* = −0.63), suggesting that inspiratory muscle training attenuated the postoperative reduction in inspiratory muscle strength. In addition to the pulmonary changes proposed by Cordeiro et al. [3], diaphragm weakness—a frequent consequence in patients requiring mechanical ventilation, which is common in the postoperative period after CABG—is associated with a significant decline in respiratory function and a higher mortality risk [21,22].

Nasser et al. [9] suggested a relationship between lung function and reduced inspiratory muscle strength. The authors speculate that the reduction in respiratory function observed eight weeks after surgery was associated with restricted chest movements caused by pain related to the surgical procedure. The declines in FEV_1_ and MEP were of moderate magnitude (Cohen’s *d* = −0.64 and −0.48, respectively), whereas the decrease in MIP was smaller (Cohen’s *d* = −0.35), suggesting a limited impact on inspiratory muscle strength. Urell et al. [10] also proposed an association between decreased inspiratory muscle strength and impaired lung function, with differences in respiratory muscle strength two months after surgery, although with effect sizes of Cohen’s *d* = −0.22 for MIP, −0.20 for MEP, and −0.27 for FEV_1_, suggesting a limited clinical impact.

Johnson et al. [16] reported pulmonary complications two months after surgery in patients who underwent coronary artery bypass grafting, showing significant postoperative reductions in FVC, FEV_1_ and MEP, that persisted for at least eight weeks with a moderate-to-large effect sizes for FVC (Cohen’s *d* = −0.78) and FEV_1_ (Cohen’s *d* = −0.76), and a small effect for MEP (Cohen’s *d* = −0.15), indicating a greater impact on lung volumes than expiratory muscle strength. Riedi et al. [17] demonstrated a significant decrease in respiratory muscle strength in the postoperative period, with a large effect size on MEP (Cohen’s *d* = −0.85) and a small effect size on MIP (Cohen’s *d* = −0.29) assessed one week after surgery. Despite differences in the timing of patient assessments, both studies reported the same type of complications. However, Johnson et al. [16] did not provide information regarding whether the patients performed any type of postoperative rehabilitation, while patients in Riedi et al. [17] study performed a physiotherapy intervention according to the standard hospital protocol.

Sacvi et al. [18] reported large postoperative reductions in the control group for FVC (Cohen’s *d* = −1.32), FEV_1_ (Cohen’s *d* = −0.88), MEP (Cohen’s *d* = −1.18), and MIP (Cohen’s *d* = −1.49). In the intervention group, which underwent inspiratory muscle training, declines were also observed for FVC (Cohen’s *d* = −1.53), FEV_1_ (Cohen’s *d* = −1.39), and MEP (Cohen’s *d* = −1.43), but MIP showed a small positive effect (Cohen’s *d* = 0.43). These results demonstrate that CABG significantly reduces pulmonary function, but that inspiratory muscle training, which was applied in the intervention group in the early postoperative period attenuated the decline of the MIP.

Baseline comorbidities play a significant role in postoperative pulmonary impairment following CABG [16,17]. Hypertension, diabetes mellitus, hyperlipidemia, smoking or alcohol history, and physical inactivity—reported in two of the included studies—compromise preoperative respiratory reserve and may exacerbate the postoperative decline in lung function. These conditions contribute to systemic inflammation and reduce diaphragmatic function, exacerbating postoperative atelectasis and respiratory muscle weakness [17,18]. Established cardiac risk scores, such as EuroSCORE II, which include respiratory comorbidities and frailty measures, were only reported in Sacvi et al. [18]. These scores could help identify CABG patients at the highest risk of postoperative pulmonary impairment and guide preoperative optimization strategies in future research [23].

The surgical procedure induces a systemic inflammatory response that affects cardiac output, lung compliance and thoracic expansion, leading to reduced functional capacity and the impairments in the respiratory and cardiovascular systems, which increase the risk of postoperative complications [3,16]. Additionally, the surgery can cause pain and alterations in chest wall mechanisms, contributing to the development of atelectasis or pneumonia, prolonging hospital stays, and delaying recovery [10,18]. Moreover, the use of mechanical ventilation—common after surgery—may result in diaphragm dysfunction, further contributing to the postoperative decline in respiratory function [21,22].

Related to the cardiovascular system, infarct size and myocardial viability may predict contractile recovery post-revascularization and influence pulmonary rehabilitation by improving cardiac output and diaphragmatic perfusion, helping select patients who benefit most from CABG [24].

To address the postoperative decline in respiratory function following coronary artery bypass grafting, several strategies have been proposed to reestablish respiratory muscle strength and improve overall functional capacity [3]. Respiratory muscle training may enhance respiratory muscle strength and contribute to the recovery of pulmonary function [3,8,25]. Incorporating respiratory muscle training into rehabilitation programs may accelerate functional recovery and promote independence in daily activities. This systematic review supports the inclusion of respiratory muscle training in standard rehabilitation protocols for patients undergoing CABG [8,25]. Evidence suggests that such strategies may attenuate the negative impact of surgery on respiratory function, promoting faster pulmonary recovery and potentially reducing postoperative complications and hospital stays [8,22]. Evidence suggests that respiratory dysfunction, exacerbated by baseline comorbidities and systemic inflammation, is associated with an increased risk of pulmonary complications, prolonged hospital stays, and elevated healthcare costs. Myocardial viability emerges as a crucial predictor of cardiopulmonary recovery, suggesting that preoperative cardiac imaging assessment can optimize patient selection and stratify postoperative respiratory risks [9,24].

## 5. Limitations

Although efforts were made to control the methodology as rigorously as possible, this systematic review has some limitations. The use of different postoperative interventions or rehabilitation protocols, as well as different times of assessment, limits the interpretation of the findings. The heterogeneity of the assessments makes it difficult to determine if the reductions in respiratory function are due to the surgery, the type of rehabilitation used, or simply the time since surgery. Further research should include other types of cardiac surgery, and well-established protocols for respiratory muscle assessment.

## 6. Conclusions

This systematic review demonstrates consistent postoperative impairments in pulmonary function following coronary artery bypass grafting, confirming a decline in respiratory muscle function, with moderate-to-large effect sizes across the analyzed variables including MIP, MEP, FVC, and FEV_1_. However, the small number and heterogeneity of the available studies limit the strength of these findings. Baseline comorbidities and the systemic inflammation related to the surgery are related to a higher incidence of postoperative complications. These findings support the clinical importance of monitoring respiratory function after CABG and incorporating respiratory muscle training into standard rehabilitation programs, to optimize health-related quality-of-life outcomes.

## Figures and Tables

**Table 1:** Search strategy and inclusion/exclusion criteria based on PECOS strategy.

PECOS	Inclusion Criteria	Exclusion Criteria	Search Term
Population	Individuals with more than 18 years of age submitted to coronary artery bypass grafting	Individuals with less than 18 years of age or submitted to other types of cardiac surgery	Adult
Exposure	Individuals submitted to coronary artery bypass grafting	Individuals submitted to other types of cardiac surgery	Cardiac rehabilitation Heart surgery Coronary heart disease Cardiac surgery Coronary artery bypass grafting GABG
Comparison	Pre- and post-surgery respiratory function comparison		
Outcome	Respiratory function	No respiratory function assessment or other outcomes evaluated	Respiratory function Respiratory muscle strength Muscle strength Inspiratory muscle training
Study Design	Original research articles	Systematic reviews, meta-analysis and editorials	

**Table 2:** Characteristics of the included studies.

Authors	Sample Size and Sex	Age	Study Design	Timing of Assessment	Respiratory Function Outcome	Main Conclusions
Cordeiro et al., 2016	Control group (25) M—16; F—9	Control Group: 57 ± 14.7	Randomized controlled trial	Preoperative and at discharge	Maximal inspiratory pressure	Significant reduction in maximal inspiratory pressure in both groups after surgery, more significant in the control group (97.5 ± 18.2 to 69.5 ± 14.9 cmH_2_O in the control group, *p* = 0.00001, and 94.2 ± 16.2 to 83.1 ± 19.1 cmH_2_O in the intervention group, *p* < 0.01).
	Intervention group (25) M—11; F—14	Intervention: 56.4 ± 13				
Johnson et al., 1996	90 patients	CABG: 65 ± 8.8	Observational study	Preoperative, at discharge and 8 weeks after discharge	Forced vital capacity Maximal expiratory volume in one second Maximal expiratory pressure	Postoperative changes in respiratory muscle strength persisted up to at least 8 weeks after surgery (FVC 3.51 ± 0.82 to 2 91 ± 0.72 L, *p* < 0.01; FEV_1_ 2.74 ± 0.74 to 2.2 ± 0.63%, *p* < 0.01; MEP 93 ± 28 to 89 ± 26 cmH_2_O, *p* > 0.01).
Naseer et al., 2019	28 patients	65 ± 7	Observational study	Preoperative and 8 weeks after surgery	Forced expiratory volume one second Maximal inspiratory pressure Maximal expiratory pressure	Significantly decreased in all outcomes observed 8 weeks after surgery, showing a relationship between a reduction in inspiratory muscle strength and lung function (FEV_1_ 2.87 ± 0.45 to 2.5 ± 0.68 L, *p* = 0.0001; MIP 81.75 ± 22.04 to 74.56 ± 18.86 cmH_2_O, *p* = 0.146; MEP 98.55 ± 22.24 to 88.86 ± 18.14 cmH_2_O, *p* = 0.019).
Riedi et al., 2010	34 patients	54.05 ± 13.6	Observational study	Preoperative and 5 days after surgery	Maximal inspiratory pressure Maximal expiratory pressure	Significantly decreased in respiratory muscle strength in the postoperative period (MEP 89.18 ± 30.18 to 66.8 ± 22.11 cmH_2_O, *p* > 0.05, and MIP 106.2 ± 49.42 to 91.5 ± 52.2 cmH_2_O, *p* < 0.05)
Sacvi et al., 2011	Control group (21) M—19; F—2	Control group: 57.48 ± 11.48	Randomized controlled trial	Preoperative and 5 days after surgery	Forced expiratory volume one second Forced vital capacity Maximum inspiratory pressure Maximum expiratory pressure	Significantly decreased in all outcomes five days after cardiac surgery in both groups, with a reduction in lung function (FVC 85.00 ± 13.71 to 66.43 ± 14.42%, *p* < 0.05, in the control group, and 88.00 ± 16.36 to 64.00 ± 14.94%, *p* < 0.05, in the intervention group; FEV_1_ 77.24 ± 14.59 to 64.29 ± 14.90%, *p* < 0.05 in the control group and 85.95 ± 16.75 to 63.73 ± 15.06%, *p* < 0.05, in the intervention group); MEP 101.71 ± 22.22 to 73.43 ± 25.52 cmH_2_O, *p* < 0.05, in the control group, and 106.55 ± 33.27 to 69.82 ± 14.60 cmH_2_O, *p* < 0.05, in the intervention group;); MIP 84.62 ± 17.26 to 57.24 ± 19.48 cmH_2_O, *p* < 0.05, in the control group and 82.64 ± 29.31 to 95.45 ± 30.32 cmH_2_O, *p* < 0.05, in the intervention group)
	Intervention group (22) M—19; F—3	Intervention group: 62.82 ± 8.69				
Urell et al., 2016	16 patients	67 ± 10	Observational study	Preoperative and 2 months after surgery	Maximal inspiratory pressure Maximal expiratory pressure Forced expiratory volume one second	Differences in respiratory muscle strength two months after surgery, although an association between decreased inspiratory muscle strength and impaired lung function was shown (MIP 78 ± 24 to 73 ± 22 cmH_2_O, *p* = 0.19; MEP 122 ± 33 to 115 ± 38 cmH_2_O, *p* = 0.018; FEV_1_ 3.0 ± 0.8 to 2.8 ± 0.7 L, *p* = 0.001)

**Table 3:** Quality assessment scores of selected studies (ROBINS-E).

	Confounding	Selection	Measurement of Exposure	Departure From Exposure	Missing Data	Measurement of Outcomes	Reported Results	Overall Bias
Johnson et al., 1996	M	M	L	M	L	L	M	M
Naseer et al., 2019	M	M	L	M	L	M	M	M
Riedi et al., 2010	M	M	L	M	M	M	M	M
Urell et al., 2016	M	M	L	M	L	M	M	M

L: low; M: moderate.

**Table 4:** Quality assessment scores of selected studies (Cochrane risk-of-bias tool 2 for randomized trials).

	Randomization Process	Deviations from Intended Interventions	Missing Outcome Data	Measurement of the Outcome	Selection of the Reported Result	Overall			
Cordeiro et al., 2016	+	?	+	+	+	+		+	Low Risk
Sacvi et al., 2011	+	+	+	+	+	+		?	Some Concerns

## Data Availability

Data sharing does not apply to this article.

## Supplementary Materials

The following supporting information can be downloaded at: https://www.mdpi.com/article/10.3390/jcm15072793/s1, Table S1: PRISMA 2020 Checklist.
